# A brief intervention for PTSD versus treatment as usual: Study protocol for a non-inferiority randomized controlled trial

**DOI:** 10.1186/s13063-021-05674-y

**Published:** 2021-10-25

**Authors:** Halvor Stavland, Camilla Refvik, Jarle Eid, Rafiq Lockhat, Åsa Hammar

**Affiliations:** 1 Faculty of Psychology, University of Bergen, Bergen, Norway; 2 Center for Crisis Psychology, University of Bergen, Bergen, Norway; 3 Cape Town, Summit Clinic, Cape Town, South Africa; 4 Department of Biological and Medical Psychology and Division of Psychiatry, Haukeland University Hospital, University of Bergen, Bergen, Norway

**Keywords:** PTSD, Brief intervention, BWRT, Treatment, Randomized controlled trial, Study protocol

## Abstract

**Background:**

Although existing treatment methods are effective in alleviating PTSD symptoms, several barriers to care exist, such as waiting times, avoidant tendencies, shame and stigma, potentially leading to fewer people seeking therapy or premature dropouts. A potential solution to battling these barriers is Brain Working Recursive Therapy (BWRT), a single-session exposure-oriented intervention for PTSD. Although not yet subjected to empirical investigation, clinical experiences suggest an often immediate and long-lasting effect following the intervention related to patient’s symptomatology and functional abilities.

**Methods:**

The current study protocol outlines a plan to conduct the first non-inferiority randomized controlled trial aimed to explore the efficacy of BWRT compared to treatment as usual (TAU), operationalized as any evidence-based trauma treatment method administered in Norwegian out-patient clinics. Eighty-two participants will be allocated at a 1:1 ratio to one of the following treatment conditions: (1) BWRT or (2) treatment as usual. Participants will be compared on several variables, including changes in PTSD symptoms (primary objective), and changes in perceived quality of life, rumination, functional and cognitive ability (secondary objective). Data collection will take place baseline (T1), within three weeks post treatment (T2) and at 6-month follow-up (T3).

**Discussion:**

Should BWRT prove to be non-inferior to treatment as usual, this brief intervention may be an important contribution to future psychological treatment for PTSD, by making trauma treatment more accessible and battling current barriers to care.

**Trial registration:**

191548, 24.05.2021. ClinicalTrials.gov PRS: Release Confirmation

## Administrative information

Note: the numbers in curly brackets in this protocol refer to SPIRIT checklist item numbers. The order of the items has been modified to group similar items (see http://www.equator-network.org/reporting-guidelines/spirit-2013-statement-defining-standard-protocol-items-for-clinical-trials/).

**Table:** 

Title {1}	A brief intervention for PTSD versus treatment as usual: Study protocol for a non-inferiority randomized controlled trial
Trial registration {2a and 2b}.	191548 Korttidsbehandling av PTSD - Studie protokoll II Regional Committee for Medical and Health Research Ethics, Western-Norway
Protocol version {3}	V 1.0
Funding {4}	University of Bergen, seed funding
Author details {5a}	*Halvor Stavland, Faculty of Psychology, University of Bergen, Norway, *Camilla Refvik, Faculty of Psychology, University of Bergen, Norway, * Shared first authorship Jarle Eid, Center for Crisis Psychology, University of Bergen, Norway, Rafiq Lockhat, Cape Town, Summit Clinic, Cape Town, SA Åsa Hammar, University of Bergen, Department of Biological and Medical Psychology and Division of Psychiatry, Haukeland University Hospital, University of Bergen, Norway
Name and contact information for the trial sponsor {5b}	N/A
Role of sponsor {5c}	N/A

## Introduction

### Background and rationale {6a}

Posttraumatic stress disorder (PTSD) is a debilitating psychiatric disorder characterized by symptoms such as intrusive thoughts and re-experiencing, avoidance of trauma-related reminders, negative alterations in cognition and mood, and marked alterations in arousal and reactivity [1]. It can develop following exposure to events of exceptionally threatening character, either in the form of a single traumatic event, or following prolonged exposure to trauma [2]. Large parts of the population will be exposed to at least one potentially traumatic event over the course of their lifetime [3], with the majority experiencing a pattern of acute traumatic reactions, followed by a path of natural recovery [4]. However, a small proportion of trauma victims will not recover from these experiences and go on to develop PTSD [5]. The cross-national lifetime prevalence of PTSD has been estimated to 3.9% in the general population, and 5.6% among those exposed to a potentially traumatic event [6]. Estimates of PTSD prevalence have also been obtained within the Norwegian population, in which a recent population study indicated a point prevalence of PTSD of 3.8% for men, and 8.5% for women in Norway [2].

Although only a small proportion of trauma victims will go on to develop PTSD, the potential consequences and associated clinical outcomes related to the disorder have proven to be severe and long lasting. In addition to the severe acute and chronic symptoms often experienced by many trauma survivors, PTSD is also associated with a high frequency of comorbid mental disorders [7], including diagnoses such as major depression, anxiety disorders, substance use disorders, psychotic disorders and borderline personality disorder [8]. Furthermore, PTSD has repeatedly been found to correlate with additional symptomatic features and severe impairments across different aspects of functioning, including higher likelihood of suicidal ideation and attempts of suicide [8], lower quality of life [9, 10], higher levels of pathological rumination [11, 12], neurocognitive impairment [13], as well as impaired functional and occupational ability [14, 15]. Similar findings have been obtained in studies investigating the impact of trauma in people with subthreshold PTSD, referring to those who experience meaningful symptoms of PTSD without meeting the full diagnostic criteria [16]. This group has been found to experience levels of comorbidity, suicidality and psychosocial impairments that are comparable to those diagnosed with PTSD [17, 18], underlining the detrimental effects that may be related to trauma exposure, and highlighting a need for treatment amongst a broad group of those battling with symptoms of PTSD.

#### Treatment methods for PTSD

Several treatment methods have been developed for PTSD, with strong empirical evidence supporting their effectiveness in reducing symptoms. According to best-practice treatment guidelines, trauma-focused treatments are highly recommended for PTSD [19, 20], referring to treatments that aim to directly target the traumatic memory, along with its associated thoughts and feelings [21]. Such methods include Eye Movement Desensitization and Reprocessing (EMDR) [22], Cognitive Therapy for PTSD (CT-PTSD) [23], Prolonged Exposure (PE) [24] and Cognitive Processing Therapy (CPT) [25], and normally requires between 8–12 treatment sessions [19]. Although based on slightly different theoretical models [26–28], an important commonality for the trauma-focused treatments is that they are partly based on an understanding of PTSD as being maintained by maladaptive processing of the traumatic memory, in which the goal of therapy is to help the patients process and reorganize their memory functions [29]. The therapies utilize slightly different strategies for processing the traumatic memories, ranging from verbal descriptions to visualization [29]. In addition, methods may vary in degree of exposure to trauma, with some focusing on the traumatic memory as a whole, while others target particular difficult moments of the memory [29]. Some trauma-focused therapies also aim to discriminate between the patients present and the traumatic event, assisting the patient to refocus their attention to parts of their life outside the trauma [29]. Despite the growing consensus on the effectiveness of these treatment methods, providing them to trauma survivors is not always feasible, and studies indicate that only a minority of those diagnosed with the disorder will seek treatment [30]. As such, a significant proportion of those who suffer from PTSD do so in the absence of appropriate treatment methods [31].

#### Barriers to care

Several barriers to care have been outlined in the literature as potentially preventing those who suffer from PTSD from receiving treatment [31]. Firstly, existing methods often require a relatively high investment of resources not available to all affected individuals. This is especially true for certain groups, such as people of low socio-economic status, refugees, and those living in warzones or underdeveloped nations, where the governmental health service and welfare infrastructure might be underdeveloped, inaccessible or costly [32, 33]. Even in developed nations, access to treatment may be difficult or slow to obtain [34], due to structural barriers such as long waiting times and insufficient treatment availability [35, 36]. Yet when such treatments are made available, other factors may prevent trauma victims from seeking help, including psychosocial factors such as shame and stigma [37]. In cases where people do seek out and receive treatment for PTSD, premature dropouts are common [38, 39], which might in part be due to the amount of time and resources required for the treatment, as it has been found that a greater number of sessions correlates with higher premature dropout rates [39]. Additionally, reasons for dropout might also be related to specific characteristics and symptoms of trauma patients, and studies have found avoidant tendencies to be associated with a higher likelihood of dropout from therapy [40]. Hence, in addition to serving as a core symptom of PTSD [1], avoidance of trauma-reminders might also function as a crucial barrier to care.

Common for the outlined barriers to care, is that they may deprive patients of the opportunity of receiving sufficient evidence-based treatment [31]**,** potentially leading to symptom exacerbation and greater burden of disease. When left untreated, PTSD tends to develop into a chronic condition, with findings indicating that more than one third of people with PTSD will continue to experience symptoms 30 years after onset of the disease [41]. As PTSD continues to add considerably to the national burden of disease both in Norway and in other parts of the world [42, 43], there is currently a need to identify alternative treatment methods that can address the existing barriers to care in order to obtain greater attendance and engagement in treatment, and successfully accommodate the needs of people suffering from symptoms of PTSD.

#### Brief treatments

Research into time- and resource-effective treatments may prove to be a valuable endeavor in the quest to provide viable additions - maybe even alternatives - to the standard treatments of PTSD. These treatment methods are commonly known as “brief treatments” and are often based on a condensed version of various long-form standard treatments [44–49]. The idea of condensed therapies for psychological disorders is well established, with the Bergen 4-Day Treatment (B4DT) [50] being one of the most promising examples of such practices. This treatment has proven to be highly effective both in and outside of Norway [51, 52], with results indicating that the treatment of severe mental illnesses does not necessarily have to be as time-consuming as previously assumed. Promising results have also been gained for brief interventions specifically developed for treating PTSD, including a five-session written exposure therapy (WET) [53], which has been found to be non-inferior to standard psychological treatment, despite its significantly reduced treatment dose [53]. Additionally, a three-session concentrated version of EMDR [48] as well as a one to five-session accelerated resolution therapy [54] have also demonstrated significant alleviation of PTSD symptoms following interventions. In addition to the promising results of these interventions, there is growing evidence supporting the notion that recommended evidence-based treatments, such as EMDR, PE and CPT, might be delivered in a more intensive manner, with results indicating improved treatment response and reduced dropout [55], underlining the potential of delivering therapy in a less time-consuming format, without compromising on treatment outcomes.

#### BWRT

A recent addition to the brief treatment arena for PTSD is Brain Working Recursive Therapy (BWRT®) [56], a single-session intervention based on an understanding of PTSD as being caused and maintained by maladaptive processing of the traumatic memory, in which the main goal of the therapy is to help the client change these maladaptive patterns in order to alleviate symptoms. The intervention is carried out following a strict protocol with a well-defined procedure [56] without the patient having to disclose the full details of the traumatic memory. In brief, BWRT moves through different stages, and utilizes visualization techniques in processing of the traumatic memory. Exposure to the traumatic memory is done in a brief manner, mainly focusing on the worst part of the traumatic memory. It also focuses on generating a new memory of the traumatic event, in which the patient is encouraged to visualize their preferred response to the traumatic situation in mind. In addition, the intervention aims to assist the patient in the discrimination between trauma and other life events, and patient is guided through visualizing a future memory in which the traumatic memory is no longer interfering with their lives.

#### Rationale for a randomized controlled trial

Although not yet subjected to systematic empirical investigation, case studies indicate that BWRT might hold the potential for achieving sustainable change related to PTSD symptoms following a single session, in a way that is well-tolerated by patients [57]. It is currently a widespread method practiced in large parts of South Africa, with clinical experiences suggesting an often immediate and long-lasting effect following the intervention, related to patient’s symptomatology, as well as their functional abilities in personal and vocational settings. However, the current lack of empirical evidence regarding the method might leave an effective and resource-efficient trauma treatment out of reach for patients and public health professionals in other parts of the world. Given the outlined challenges related to existing trauma treatment methods, as well as the growing evidence supporting the potential of brief interventions, BWRT could serve as a positive addition to the future of trauma treatment. Considering its properties, BWRT might also be eligible to battle some of the existing barriers to care, and thus make trauma treatment more widely accessible, easier to administer on a larger scale, less time-consuming, and better tolerated by patients. The current study protocol outlines a detailed plan to conduct the first non-inferiority randomized controlled trial aimed to explore the efficacy of BWRT compared to treatment as usual (TAU), and thus represents an important step towards empirically investigating the efficacy of this brief treatment method. Specifically, we wish to examine whether BWRT is non-inferior to TAU, despite its compromised timeframe.

### Objectives {7}

#### Primary objective

The primary objective of the proposed study is to measure changes in severity of PTSD symptoms (primary outcome), from 1-week pre-intervention (T1) to within 3 weeks post-intervention (T2), and from T2 to 6 months follow-up (T3), and to compare the results of the two groups.

#### Secondary objectives

As our secondary objectives, we plan to explore any potential differences between the BWRT and TAU groups on self-reported measures related to participants’ perceived quality of life, levels of rumination, functional and cognitive ability (secondary outcomes) following the interventions (T1 to T2 and T2 to T3).

### Trial design {8}

This study is a two-armed non-inferiority randomized controlled trial with a parallel group design. Participants will be allocated at a 1:1 ratio to one of the following treatment conditions: (1) BWRT or (2) treatment as usual.

## Methods: Participants, interventions and outcomes

### Study setting {9}

The study will take place in Bergen, Norway. Interventions will be carried out at the Department of Biological and Medical Psychology, University of Bergen, as well as the Centre for Crisis Psychology.

### Eligibility criteria {10}

**Participants.** Based on a statistical power analysis described under the statistics sections, the study sample will consist of 82 adults between the ages 18–65. To be eligible to take part in the study, participants must have experienced at least one traumatic experience throughout their lifetime. In addition, participants must meet the DSM-5 criteria for PTSD [1], or subthreshold PTSD. In reference to the majority definition for subthreshold PTSD [16], the following DSM-5 criteria must be fulfilled: A (exposure to traumatic stressor), in addition to three out of four of the following symptom-clusters; B (intrusion), C (avoidance), D (negative alterations in cognitions and mood), or E (alterations in arousal and reactivity). In addition, a marked decline in functioning and symptom persistence for over a month is also required. To ensure a firm ecological validity and greater generalizability of our results, we plan to include a study sample with a heterogeneous trauma history. Thus, participation will not be restricted to one particular type of trauma (e.g. sexual violence). In addition, common comorbid conditions such as anxiety and mild to moderate cases of depression will not be reasons for exclusion. All comorbid conditions will be registered and recorded at inclusion and reported in our results.

#### Exclusion criteria

Participants who meet any of the following criteria will be excluded from the study: (1) ongoing psychotic disorders, or a history of psychosis, (2) severe suicidal ideation, (3) bipolar disorder*,* (4) BMI index too low to benefit from psychological interventions, (5) severe alcohol or substance dependence, (6) serious somatic illness or brain damage, (7) participation in concurrent psychotherapy. These criteria have been chosen to optimize participant safety, and to ensure that the results of the study are both accurate and meaningful.

#### Dropout criteria

Participants whom for whatever reason discontinues their participation in the study, will be registered and reported in our results as dropouts. If available, reason for drop-out will be reported. All data related to the participant’s participation will be deleted unless the participant consents to the continued use of existing data.

#### Eligibility criteria for psychotherapists

All psychologists carrying out interventions are required to have at least one year of clinical experience. Treatment as usual will be carried out by clinical psychologists affiliated with the University of Bergen. The BWRT intervention will be carried out by clinical psychologists who have already completed an intensive 2-day BWRT-workshop in South Africa, and hence are certified in the BWRT method.

### Who will take informed consent? {26a}

Informed consent will be obtained by the project coordinator, or available study staff.

### Additional consent provisions for collection and use of participant data and biological specimens {26b}

Not applicable, as no ancillary studies are planned.

## Interventions

### Explanation for the choice of comparators {6b}

The aim of the proposed study is to investigate whether BWRT is noninferior to current evidence-based therapies “as practiced”. In order to investigate this matter, we have chosen “treatment as usual” as a comparator to BWRT, with the relatively open-ended definition of TAU as any evidence-based therapy for PTSD provided within the Norwegian health care system and adhering to the National Institute for Health and Care Excellence (NICE) guidelines for treating PTSD [19]. The rationale behind choosing this comparator in the proposed study, is that we consider it as a necessity to compare the efficacy of BWRT to those treatment options that would be available in absence of the brief intervention. Additionally, “treatment as usual” as a comparator positively impacts the feasibility of the study, as there will be no need to recruit expert clinicians of a specific therapy method or rigorous training of therapists to ensure treatment protocol adherence. Hence, the current design allows for a feasible comparison of the efficacy of BWRT compared to the general high standards of practice in PTSD-treatment in Norway and enables the investigation of whether a one-hour session of BWRT can come close to the effects of the multifaceted, individually adjusted and resource-intensive PTSD-treatment currently residing in the Norwegian health care services. Given that the empirical investigation of BWRT’s efficacy is still in its infancy, we consider TAU as a feasible basis of comparison in the current non-inferiority trial, in order to inform whether BWRT offers real therapeutic remedies for trauma patients, in a way that is non-inferior to existing treatment methods.

### Intervention description {11a}

#### BWRT

The BWRT [56] intervention will be carried out in a single session, with a duration of approximately 55 minutes. In accordance with BWRT’s understanding of PTSD as being caused by maladaptive processing of the traumatic memory, the intervention begins by implementing aspects of psychoeducation, in order to socialize the patient to this understanding. This first step involves the use of analogies, by metaphorically comparing the traumatized brain to a computer with malware, representing the PTSD-symptoms, that is currently causing impaired functioning. The treatment is further described to the patient as working directly on this malware, in order to regain functioning and hence reduce symptoms and distress. Following this initial explanation of the coming treatment process, the intervention moves through several different phases, using a strict but simple protocol, focusing on the patient's past, present and future. The intervention can be described as a therapist-assisted exposure, in which elevated levels of arousal are seen as a window to intervene.

*Visualization.* Initiating the treatment, the patients will be instructed to close their eyes, and imagine their worst traumatic memory, without any form of verbal recollection. A crucial part of the therapy is emotional involvement, in which the patient is instructed to notice what they are feeling, and where they are feeling it. The clinician is looking for high emotional involvement during this phase, in which the patient is asked to report, on a scale from 1 to 10, how distressing the conjured image is. If the patient rates an 8 or higher, or it is obvious based on body language that the memory is severely upsetting, the clinician asks the patient to quickly “zoom”' into the worst moment of the traumatic memory and indicate, by raising a finger, when they have it in mind. The therapist then claps her hands and asks the patient to freeze the image in mind. What follows is then a series of mental imagery instructions where the patient is asked to change the traumatic memory in any preferred way that makes the patient feel better. The patient is then instructed to focus on where they are now, by grounding themselves to where they are sitting. Following this step, the patient is then instructed to create a positive memory of oneself in the future, visualizing being in a place where one has completely overcome the traumatic event.

*Looping.* These different stages of visualization are followed by a looping process, in which the therapist uses a script in instructing the patient to quickly move from the frozen traumatic memory, onto their preferred memory, to where they are now, moving along to the good memory of themselves in the future. This process is repeated 6 times, in which the therapist gives the instructions with high speed and intensity, and the goal is to occupy the patient’s working memory.

*Consolidation.* Following the looping process, the patient is instructed to open their eyes, and the therapist initiates a momentary chat about mundane topics, lasting for about 1–3 minutes. This is believed to be the consolidation phase, where the new memories are allowed to be unconsciously processed while the mind is occupied with simple social interaction.

*Check phase.* At the end of the session, the patient is asked to visit the original traumatic memory and report once again, on a scale from 1–10, how upsetting this memory is now. If the patient reports an activation level of 3 or more, parts of the process are repeated. Otherwise the treatment is finished by clarifying potential questions or explaining common effects of the therapy. The protocol is not published and is currently only available to licensed therapists who have been trained in the method. For a full and detailed description of the process, please contact the authors.

#### TAU

Participants who are allocated to the TAU group will be referred to treatment provided by clinical psychologists in the Norwegian health care system, who are affiliated with the Centre for Crisis Psychology. Treatment length will be capped at twelve sessions within a period of 16 weeks, in order to account for missed sessions. Therapists are free to choose whatever preferred treatment method for PTSD, as long as the selected method adheres to the NICE guidelines [19].

### Criteria for discontinuing or modifying allocated interventions {11b}

Participants are informed of the voluntary nature of participation in the study and may choose to withdraw their participation consent at any time. All cases of discontinuation will be reported to the project coordinator. Additionally, an assessment of outcome data will be carried out for the first 15 participants in the BWRT group, by an independent analyst. Should the results from such an assessment indicate that this group shows either no change or worsening of symptoms post intervention, the trial itself will be discontinued. In order to control for the limited, but existing test-retest and interrater reliability issues related to the CAPS-5 [58] no change will be defined as less than or equal to a 4-point reduction in total severity score. Should that be the case, every participant in the BWRT-group will be offered admission into the TAU-group if they wish so. Every new and existing participant in the TAU-group will hence be allowed to finish their treatments, but their participation in data collection will be discontinued.

### Strategies to improve adherence to interventions {11c}

The proposed study will implement several strategies in order to improve general adherence to interventions, focusing both on aspects related to the participants, therapists, protocol and data collection.

#### Participant adherence

In order to increase adherence to data collection procedures, general investment in the study, as well as decrease the risk of dropout, all participants will be matched with a designated staff member who will serve as their contact person. This person will be responsible for reminding participants of data collection appointments, in addition to being available for answering questions and concerns the participants might have related to the study.

#### Clinician protocol adherence

As the TAU condition involves treatment in a Norwegian clinic part of the public health care system, this group of clinicians do not need any strategies to improve protocol adherence, as this is already endemic to the system. However, they will be required to regularly report progress to the project coordinator, in order to inform timing of data collection. There will also be organized an information channel where the therapists can communicate with the project coordinator should there be any questions before, during or after the intervention period. In order to improve adherence to the BWRT protocol, additional training and videotaped “mock-therapies” without real participants will be organized for the BWRT-therapists. There will also be arranged regular meetings with the project coordinator to discuss potential obstacles, questions or complications related to protocol adherence. One therapist will be assigned a lead therapist role, with responsibility for the communication between therapists and the project coordinator outside of these regular meetings.

#### Data collection adherence

In order to reduce measurement biases, independent raters blinded to group allocation will be used to conduct all clinical interviews and non-self-report-measures related to the primary and secondary outcomes. Because independent raters are uninvolved with any other part of the study and blinded to the study design, we believe the risk of data pollution through confirmation bias will be reduced, strengthening the validity of the outcome data. To ensure that data collection will be administered safely and on schedule in a predictable manner for all parties involved, the most scientifically and assessment-experienced independent rater will serve as senior independent rater. This person will have a lead role and main responsibility for data collection scheduling, follow-ups and participant safety (during data collection) and will report directly to the project coordinator. Additionally, all independent raters will go through a training program to enhance measurement reliability, including the administration of “mock” sessions which will be videotaped and evaluated by the senior independent rater. All independent raters will report and submit their data to the senior independent rater.

### Relevant concomitant care permitted or prohibited during the trial {11d}

All participants will be asked to report any and all possible concomitant treatments during the study, including “alternative treatments” such as healers, coaches etc. Any findings will be reported to the project coordinator and dealt with accordingly should there be any suspicion of data pollution. Psychopharmacological treatment that is initiated and considered stable before the onset of participation, such as antidepressants, anxiolytics and stimulants will be permitted. If these medications are discontinued or modified in their use during the study, this will be reported and used as a variable. Any psychiatric or PTSD-specific psychopharmacological concomitant treatment will result in removal from the dataset, unless such care or treatment is acute and clearly unrelated to the treatment received during the study. Exclusion from the dataset due to concomitant care will under no circumstances exclude any participants from finishing their treatments should they wish to.

### Provisions for post-trial care {30}

All participants who wish so, will have the option of contacting the project coordinator for a referral for treatment in the Norwegian healthcare system following the study period. This information will be recorded and reported in the results.

### Outcomes {12}

In order to investigate the potential utility of the interventions, all participants will be assessed with both clinical and self-report measures. This includes monitoring the core symptom load of PTSD, as well as changes in self-reported measures of quality of life, rumination, and functional and cognitive ability, which have all been found to represent central aspects of PTSD [9–15].

#### Primary outcome measure

Potential changes in severity of PTSD symptoms from baseline to post-treatment and 6 months follow-up will be assessed using The Clinician Administered PTSD Scale for DSM-5 (CAPS-5) [58]. CAPS-5 is a structured diagnostic interview and is considered the gold standard for assessing symptoms of PTSD [59]. The interview consists of 20 questions related to each of the 4 symptom clusters defined in the DSM-5 criteria for PTSD [1]. Symptom severity is based on a combined evaluation of frequency and intensity of each individual symptom, which is assessed on a 5-point rating scale, ranging from 0–4 (0 = asymptomatic, 1 = mild/subthreshold, 2 = moderate PTSD/above threshold, 3 = severe/markedly increased, 4 = extreme) [58]. A total severity score for PTSD symptoms will be established based on the summed severity of each symptom, with scores ranging from 0–80.

Prior to the CAPS-5 baseline measure, the Life Event Checklist for DSM-5 (LEC-5) [60] will be administered to identify the presence of a potential traumatic life event **(**DSM-5 criterion A) [1]. The LEC-5 is a self-report measure consisting of 16 potentially traumatic events, in which participants indicate level of exposure related to each of these on a 6-point nominal scale [60]. It also includes one additional item in order to assess any other potentially traumatic life events not included on the measure. Based on the participant’s answers, the single worst traumatic incident, if more than one, will be identified and used as the reference trauma for the CAPS-5 measure. If there are several traumas which are strongly related to each other, for example three traumatic combat experiences during the same employment, these will be grouped as one trauma. LEC-5 will also be used to record the type and number of traumas for use in subgroup-analysis to see whether this impacts the outcomes.

#### Secondary outcome measures

The Satisfaction with Life Scale (SWLS) [61] will be used to measure potential changes in participants’ self-reported quality of life. The SWLS consist of 5 statements related to global life satisfaction and subjective well-being, using a 7-point scale to indicate agreement (from 1 – strongly disagree, to 7 – strongly agree), and with scores ranging from “extremely dissatisfied” to “extremely satisfied”. The SWLS has shown sufficient sensitivity in detecting potential changes in life satisfaction over the course of clinical interventions [62] and will be handed to participants at baseline, post-intervention, and at the 6-month follow-up.

In order to measure potential changes in participants’ functional ability in work and social settings, we will be using the Work and Social Adjustment Scale (WSAS) [63]. The WSAS assesses participants’ self-reported degree of impairment related to five domains of functioning (ability to work, home management, social leisure activities, private leisure activities, close relationships), consisting of one statement related to each domain, with a response scale indicating perceived impairment on a scale from 0 (not at all) to 8 (very severely). The WSAS is considered a reliable and valid measure of work and social adjustment [63], and participants will be asked to fill out the questionnaire at baseline, post-intervention, and at the 6-month follow-up.

Potential changes in cognitive functioning will be assessed using the Perceived Deficits Questionnaire-Depression, 5-item (PDQ-D5) [64]. The PDQ-D5 is a 5-item questionnaire, assessing the presence of problems related to memory, attention and concentration during the past week. The PDQ-D5 uses a 5-point Likert scale, with answers ranging from 0 (never in the past 7 days) to 4 (very often [more than once a day]). Participants will be asked to fill out the PDQ-5D at baseline, post-intervention and 6-month follow-up.

Potential changes in participants levels of rumination will be measured using the Ruminative Responses Scale (RRS) [65]. The scale consists of 22 statements related to ruminative tendencies, using a 4-point Likert scale to indicate frequency of different types of ruminative thinking, with responses ranging from 1 (almost never) to 4 (almost always). The questionnaire will be handed to participants in both groups at each point of data collection (T1-T3) in order to investigate potential differences between the two groups in ruminative thinking from baseline to post-intervention and 6-month follow-up.

### Participant timeline {13}

An illustration of the central timepoints of the study is provided in Table 1.

**Table 1:** Schedule of enrolment, interventions, and assessments. Recommended SPIRIT item. The Mini-International Neuropsychiatric Interview; M.I.N.I, The Clinician Administered PTSD Scale for DSM-5; CAPS-5, BrainWorking Recursive Therapy; BWRT, treatment as usual; TAU. Template retrieved from: http://www.spirit-statement.org/publications-downloads/

	STUDY PERIOD				
TIMEPOINT**	Enrolment	Allocation and baseline assessment	Post-allocation		6-month follow-up
			**Treatment**	**3 weeks post-treatment**	
**ENROLMENT:**					
Eligibility screen	X				
Informed consent	X				
*M.I.N.I.*	X				
*Demographic information*		X			
*CAPS-5*		X			
Allocation		X			
**INTERVENTIONS:**					
*BWRT*			X		
*TAU*			X		
**ASSESSMENTS:**					
*CAPS-5*				X	X
The Satisfaction with Life Scale (SWLS)		X		X	X
Work and Social Adjustment Scale (WSAS)		X		X	X
Rumination Response Scale (RRS)		X		X	X
Perceived Deficits Questionnaire-Depression, 5-item (PDQ-D5)		X		X	X

### Sample size {14}

#### Power analysis

The following formula provided in Flight and Julious [66] was used to perform a power analysis to determine the required sample size for the primary study aim to test non-inferiority based on CAPS-5 symptom severity:

$$ n=\frac{\left(r-1\right){\left({Z}_{1-\beta }+{Z}_{1-\alpha}\right)}^2{\sigma}^2}{r{\left(\left({\mu}_A-{\mu}_B\right)-{d}_{N1}\right)}^2} $$

Following previously published non-inferiority trials [44, 67], we used 20 as an estimate of the population standard deviation on the CAPS-5 and a non-inferiority limit (dN1) of 10. Differences of less than 10 between TAU and BWRT on the CAPS-5 are thus considered clinically non-significant. Given that the BWRT group will receive a considerably shorter treatment than TAU, it can be argued that there may be a small difference in scores between treatments. We therefore set μA – μB = 1 (10% of the non-inferiority limit of the mean difference). The remaining specifications were power (1-β) of .80, a Type I error rate (α) of .05, and an equal allocation *(r)* to the two treatment groups. With these specifications, 41 participants are required per treatment group.

### Recruitment {15}

Participants will be recruited through a multifaceted recruitment strategy, including recruitment brochures to general practitioners, the student psychological welfare services, targeted social media ads, as well as posters and leaflets distributed throughout the city of Bergen. Recruitment will also take place through notifying the surrounding government funded out-patient clinics, which due to their amount of referrals is expected to be the main recruitment channel. Recruitment methods will include a brief description of the study, in addition to contact information. Evaluation of recruitment strategies will be done continuously to maximize likelihood of achieving adequate enrolment, and recruitment strategy for each enrolled participant will be recorded and reported in the results. Additionally, a diagram of participant flow throughout the trial will be provided, including information regarding how many were assessed for eligibility, excluded due to declining to participate or meeting exclusion criteria, randomized and allocated to interventions, lost to follow-up or discontinued interventions, completed the trial and were included in analysis, and how many who were excluded from analysis.

#### Screening procedure

Prior to inclusion, potential eligible participants who have expressed interest in participating will be contacted by study staff and given a detailed description of the study and the estimated time frame of participation. A brief phone screening will be conducted to establish whether the individual has experienced a traumatic event, and if any of the core symptoms of PTSD has been present in the last month. For this matter, we will be using The Primary Care PTSD Screen for *DSM-5* (PC-PTSD-5) [68], a five-item screen designed to identify people with probable PTSD. A brief assessment will also be conducted for all inclusion/exclusion criteria, in which the participant will be asked about the presence of symptoms related to psychosis, bipolar disorder, and substance abuse.

#### Assessment of psychiatric comorbidity

In order to determine any comorbid psychiatric conditions of relevance to this study (see SPIRIT item 10), we will use the Mini-International Neuropsychiatric Interview (M.I.N.I) [69] during the initial assessment session. The M.I.N.I. is a brief structured interview, designed to screen for current and lifetime mental health disorders, in both accordance to the DSM-5 and ICD-10 [69]. The interview will be conducted in its totality, with exception of the PTSD module.

#### Inclusion

Based on the information provided in the screening, individuals who seem eligible to participate will be invited to an appointment at the study site where they will be provided with thorough information regarding the study and voluntary nature of participation. Individuals who wish to participate will have to fill out an informed consent form prior to further assessments. All participants will undergo diagnostic procedures prior to inclusion, conducted by an independent assessor within a week prior to onset of intervention, in which final inclusion will depend on the participants' scores on the CAPS-5 measure reaching at least subthreshold PTSD.

#### Safety procedure

Individuals who do not meet the criteria for inclusion, and hence are considered ineligible to participate in the study, will be advised to seek out their GP for further assessment. Appropriate referrals will be made for individuals who show clinically significant symptoms of PTSD, but who are excluded from the study sample due to exclusion criteria. If any of the candidates express an acute risk of suicidality, they will immediately be referred to emergency care and followed there by a member of staff.

## Assignment of interventions: allocation

### Sequence generation {16a}

After providing informed consent and completing baseline measures, all eligible participants will be randomly assigned to one of two treatment conditions: *(1) BWRT or (2) TAU.* Randomization will be carried out on a 1:1 allocation ratio, and stratification will be applied for gender (male and female) and age group (under 30, 30–45, 45–65) to ensure a greater balance within and between the two groups.

### Concealment mechanism {16b}

Participants will be randomized using a randomization software. To conceal intervention allocation, the computer-generated randomized allocation sequence will be stored in opaque sealed envelopes until both primary and secondary baseline measures have been completed.

### Implementation {16c}

The randomization process, enrolment and allocation to interventions will be carried out at the University of Bergen, by administrative staff with no involvement in the study. Staff responsible for collecting outcome measures are not allowed to receive any information regarding group allocation.

## Assignment of interventions: Blinding

### Who will be blinded {17a}

Due to the nature of the study, neither participants nor therapists can be blinded to treatment allocation. To minimize the risk of bias and measurement errors, all repeatable measures that will be used to assess the effect of treatment will be administered by independent raters (IR) who are trained in their use. Instruments intended to screen for exclusion criteria will also be performed by independent raters to avoid any recruitment bias. All IR will be blinded to the participants group allocation, and participants will be advised not to share any information regarding group allocation. Study personnel conducting the statistical analyses will also be blinded to group allocation.

### Procedure for unblinding if needed {17b}

Given that both participants and therapists are already unblinded to treatment allocation, a procedure for further unblinding is not considered applicable to the proposed study, as we foresee no situations in which unblinding of independent raters or staff working with statistics will be necessary.

## Data collection and management

### Plans for assessment and collection of outcomes {18a}

Data will be collected from clinical interviews and self-report questionnaires. Detailed information regarding primary and secondary outcome measures is provided under SPIRIT item 12. Data collection will take place at the following time points: at baseline within 1 week prior to intervention (T1), within 3 weeks post-intervention (T2) and at 6 months follow-up (T3). Information regarding demographic variables and baseline measures will be collected at the study site, at the University of Bergen. In order to ensure the completion of follow-up measures, participants will be given the option of completing such measures through an online channel, see SPIRIT item 18b.

### Plans to promote participant retention and complete follow-up {18b}

In order to promote participant retention and completion of follow-up, the same strategies as described under SPIRIT item 11c under heading “Strategies to improve adherence to interventions” will be used. In addition, the participants will be given the option of meeting with the independent rater through online video-chat channels, in order to lessen the burden of having to show up at the study site. Should the participant discontinue their treatment or if any other deviations from intervention protocols occur, the following data will be collected, if applicable: (1) description and date of deviation from intervention protocol, e.g. no-shows, non-adherence, non-compliance etc., (2) date of discontinuation, (3) reason for discontinuation, (4) baseline measures results. In case of withdrawal from informed consent, all data will be erased and only dropout and date of withdrawal will be recorded.

### Data management {19}

Self-report data will be obtained by use of electronically adapted versions of the original paper forms, through the use of a licensed provider commonly used by universities in Norway for research purposes. Data management procedures will follow the national guidelines and regulations for information security and privacy in health care services. The data will be stored for three years after study completion, before they will be fully deleted. Data integrity will be ensured by various means, including referential data rules, range checks, valid values and consistency checks. Should there be a need to re-check any of the forms, this will go through the project coordinator. Any modifications to the dataset will be documented appropriately.

### Confidentiality {27}

Raw data will be stored securely in a locked storage in a limited access area separate from personal data and plotted into the institutional server for further analyses. Data will be stored in UiB’s server SAFE for processing sensitive personal information in research. All forms related to data collection will be identified with a coded ID, and only the project manager will have access to the key that connects ID numbers to identifying information.

### Plans for collection, laboratory evaluation and storage of biological specimens for genetic or molecular analysis in this trial/future use {33}

Not applicable, as there will be no collection of biological data in the study.

## Statistical methods

### Statistical methods for primary and secondary outcomes {20a}

The two groups will be compared on all measures. All analyses will be conducted following the conclusion of data collection, using IBM SPSS Software® version 27.

#### Primary outcome analyses

Data will be treated according to the intent-to-treat principle, in which all participants will be analyzed as randomized, regardless of discontinuation or missing data. In order to triangulate the results and reduce the chance of false positives and negatives, an additional per-protocol (PP) analysis will be performed, in which only the non-deviant participants from the ITT analysis will be included. Such analyses will be conducted and reported separately. An unpaired *t*-test will be conducted in order to measure the difference between the two groups in respect to changes in severity of PTSD symptoms, as measured by CAPS-5, following treatment. In line with previous research utilizing non-inferiority designs for PTSD treatments using CAPS as a measure [53, 70], our primary analysis will operate with a non-inferiority margin of 10 total severity points. Thus, we will test whether the BWRT and TAU groups’ total severity score averages deviate more than 10 points from each other on the upper limit of a 95 % confidence interval. While the current design both ensures and aims for a high degree of ecological validity, we also plan to compare both treatment groups to a virtual control group with a set clinically informed margin of a total severity score of 20 % below average baseline measures. This imposition is to control for unmeasurable placebo and non-clinical concomitant care effects. Thus, in order to postulate non-inferiority based on our analyses, the treatment outcome averages cannot deviate more than 10 points (upper limit of 95 % CI) from each other, while both conditions will have to exceed the average baseline total severity score by 20 %. Should neither of these criteria be met, non-inferiority will not be considered achieved. In this case, subgroup analyses outlined in SPIRIT item 20b will be utilized.

#### Secondary outcome analyses

All secondary outcomes will be analyzed with linear mixed models for repeated measures to test all changes in secondary measures from T1-T2 and from T2-T3**.** Christensen & Mendoza’s formula for reliable change index (RCI) [71] will be used for significance testing and the calculations will further be categorized as “reliable change”, “no change”, or “deterioration”. To compare the two groups, an unpaired *t*-test will be executed for each secondary measure. Non-inferiority will not be assumed or tested for secondary outcomes, instead the results will be analyzed and reported as effect sizes (Cohen’s *d*).

### Interim analyses {21b}

Due to the utilization of a non-statistical stopping guideline described under SPIRIT item 11b, no advanced interim analyses will be planned. However, after the first 15 participants in the BWRT group have received the intervention, a simple pre- and post-intervention comparison of their average total severity scores as measured by CAPS-5 will be executed. This simple interim analysis will be used to inform the stopping guideline detailed under section “criteria for discontinuing or modifying allocated interventions”. The analysis will be executed blindly by an independent analyst not involved with the project.

### Methods for additional analyses (e.g. subgroup analyses) {20b}

In the case that the results of the primary outcome analyses indicate that BWRT is inferior to TAU, we plan to execute subgroup analyses based on trauma characteristics. Subgroups will be categorized according to whether the participants’ CAPS-5 reference traumatic event is of sexual or violent nature, and whether the traumatic event was witnessed or directly experienced. Additional or different categories might be used based on the make-up of the recruitment sample, but to avoid data dredging they will only relate to the central traumatic event characteristics as measured by the CAPS-5 and LEC-5. The subgroups of each intervention group will then be analyzed in the same way as in the primary outcome analysis and be tested for non-inferiority between BWRT and TAU. The rationale behind these analyses is that it allows us to see whether BWRT still might hold a potential for a certain type of trauma patient, despite showing inferiority to TAU in general.

### Methods in analysis to handle protocol non-adherence and any statistical methods to handle missing data {20c}

Analyses will be performed “as randomized” regardless of adherence for the intent to treat analysis, and clear criteria for per-protocol analysis will be employed and reported. Currently, we plan to define per-protocol as compliance with full treatment for the BWRT-condition, and attendance of 12 sessions for the TAU-group, unless clinician or participant reports the reason for fewer sessions as due to successful therapy achieved before the 12th session. Reasons for drop-out, when available, will be recorded and reported separately for the two randomized groups, followed by a qualitative analysis for comparison. This information will be used to inform appropriateness of the analyses, as well as how to handle missing data. Although subject to change based on drop-out information, our current strategy for missing data (most likely to consist of withdrawal from follow-up measurements and no-shows) is to use multiple imputation by chained equations [72].

### Plans to give access to the full protocol, participant level-data and statistical code {31c}

No later than two years following the T3-measurements, a de-identified dataset together with the statistical code will be released in Bergen Open Research Archive (BORA) with public access.

## Oversight and monitoring

### Composition of the coordinating centre and trial steering committee {5d}

Due to the scope of the study and available resources, there are currently no plans for a formal coordinating centre or trial steering committee.

### Composition of the data monitoring committee, its role and reporting structure {21a}

Due to the scope of the study and available resources, there are currently no plans for a formal data monitoring committee.

### Adverse event reporting and harms {22}

Adverse events may occur during the study, both as harms related to the intervention itself, and to the procedures of the study. While promising several advantages, there are also potential harms to BWRT as with any form of psychotherapy. Although BWRT does not require the same amount of verbal engagement as other types of trauma-focused therapy, it does require participants to engage in a vivid mental imagery “exposure” recall their worst traumatic memory whilst simultaneously keeping a high level of arousal, which might be unsettling and destabilizing to some participants. However, BWRT offers some safeguarding against this in that the protocol prepares the participant on what is going to happen, and the method does not force the exposure if the participant is not willing to. One may also argue that the mental exposure is no more harmful than the effects of flashbacks, memories and dreams about the trauma commonly associated with PTSD. Despite some studies indicating that participants in trauma-focused therapies are less likely to experience adverse events compared to those in a waiting list control, even in severely ill populations [73, 74], working with traumatic memories might still hold the potential of re-traumatization and symptom exacerbation.

In order to ensure participant satisfaction and security, all participants will be briefed concerning potential side effects, and provided with information on how to contact the project coordinator should there arise any complications, questions, or experiences of adverse effects related to the study or treatment. Additionally, the number of repeated measures has been carefully chosen to ensure participant endurance, without compromising the reliability of our research findings. If a participant at any time experiences fatigue or becomes overwhelmed, breaks will be introduced, and the participant will have the option of splitting the assessments into two different days. In addition, all independent raters are certified clinicians and will be able to assist should the participant have any need for support during data collection. Before and after each intervention or measurement the participants will be asked whether any potential adverse effects have been encountered. All unexpected or otherwise adverse events of non-acute nature observed by the project collaborators will be reported to the project coordinator and dealt with accordingly. Plans for acute emergencies involve referral to the emergency unit which is standard procedure in Norway when immediate attention is needed. As an added safety procedure, everyone in the study staff who at any point will be in direct contact with the participants will be required to be a registered mental health professional, including the independent raters.

### Frequency and plans for auditing trial conduct {23}

Due to the scope of the study and available resources, there are currently no plans for formal auditing of trial conduct.

### Plans for communicating important protocol amendments to relevant parties (e.g. trial participants, ethical committees) {25}

Protocol amendments and modifications, if any, will be done in accordance with local scientific and health regulations. Substantive modifications to protocol, if any, will be communicated to all relevant parties by the project coordinator who is responsible for all changes to protocol as well as for obtaining approval from the Reginal Committees for Medical and Health Research Ethics (REC). Minor administrative modifications or clarifications that have no effect on study conduction will go through the project coordinator and a notice will be sent to REC. All modifications, however small, will be added to a memorandum upon completion of the study.

### Dissemination plans {31a}

There will be no publication restrictions connected to the dataset. Trial results will be summarized and conveyed in text format to all participants, clinicians and independent raters. A similar report of the results will be submitted to the Norwegian Psychological Associations journal, Tidsskrift for Norsk Psykologforening. An English translation of this report will be submitted to the Psychological Society of South Africa. A simpler version of this report, meant for non-professionals, will be distributed to appropriate mental health- and patient organizations. In addition, the results will be used to submit a full scientific article to appropriate international journals with peer review, independent of results. This is especially important because the BWRT-method is widely used in South Africa, and a negative result is crucial to inform its further use, amendment or discontinuation in clinical practice. Lastly, the results will be published in BORA, owned and run by the University of Bergen.

## Discussion

Although several evidence-based treatment methods have been developed for PTSD, a significant number of those who suffer from the disorder do so in the absence of appropriate treatment [31]. Several barriers to care have been outlined as potential mediators in this asymmetrical relationship [32–40], in which a growing pool of research suggest that brief methods might hold the potential of reducing such barriers whilst still alleviating symptom severity [44–54]. Consequently, there are sufficient and promising incentives for brief treatments, such as BWRT, to be empirically investigated, despite their concentrated form. Given the increasing use of the BWRT method, we consider it an important task to gather information regarding the efficacy of the method. If BWRT as a one-session-treatment delivers results comparable to the outcomes of TAU, then there is indeed a strong and compelling case for establishing BWRT as a runner-up for future trauma treatment research. It is equally important to communicate the results of the study should the treatment show non-significant results, due to its existent use in clinical practice, as patients deserve to receive treatment that holds the potential of reducing symptomatology significantly.

As the BWRT method is still in its infancy, little knowledge exists regarding its mechanisms of change. Although considered a new treatment method for PTSD, it may be argued that BWRT holds many similarities to other trauma-focused treatments, such as EMDR, PE, CT-PTSD and CPT [22–25]. Like these methods, BWRT is also thought to work through directly targeting the patient's traumatic memory, along with its associated thoughts and feelings. Exposure to the traumatic memory is done in a brief manner, using visualization techniques, which can be seen as a similar to the techniques of EMDR [22]. Additionally, BWRT does not require the patient to fully disclose details of the traumatic event to the therapist, which is also a key element in written exposure therapy [53]. As such, BWRT does not introduce brand new concepts of trauma treatment, but instead utilizes already existing mechanisms of trauma therapy in a condensed and concentrated version. However, it might be argued that the properties of BWRT makes it especially suitable to battle existing barriers to care, in ways that may provide treatment to a greater number of trauma survivors. Should our results indicate that BWRT is non-inferior to TAU, this might have profound implications both on a systemic and an individual level.

### Implications

#### Implications on a systemic level

BWRT’s potential of providing symptom alleviation following a single session, yields promising possibilities for reducing long waiting times. Hence, by not requiring a high investment of resources, waiting times for treatment may be dramatically reduced. BWRT also represents a potential for making PTSD treatment more widely accessible through lowering the cost of treatment, which may be especially impactful in economically underdeveloped nations where psychological treatment is currently scarce [32, 33]. In this way, BWRT may represent an important step in making psychological treatment a natural part of healthcare services worldwide, providing treatment to trauma survivors on a larger scale.

#### Implications on the individual level

In addition to making treatment more widely accessible on a systemic level, BWRT might also have profound implications for individuals suffering from PTSD. This might especially be the case for those currently faced with barriers to care such as avoidance tendencies, stigma and shame [37, 40]. BWRT’s non-reliance on disclosure of details may be an attractive alternative to these patients, who might otherwise dropout or avoid seeking treatment. Hence, given the properties of BWRT, it might hold the potential of being a better tolerated method by patients compared to current treatment methods. Engagement in traditional therapies often involves a repetitive and sustained level of exposure to anxiety-provoking emotions and details of the trauma [29]. BWRT involves a quicker and less taxing exposure, this might make the treatment more emollient, and potentially serve to lower the bar for seeking additional or future therapy.

#### Implications for therapists

BWRT may have important implications for therapists through being an easily adapted and implemented tool of treatment. Every psychological treatment requires requisite and basic knowledge of psychotherapy and psychopathology. However, unlike many traditional treatment methods for PTSD, BWRT’s protocol is uncomplicated and easy to learn and adhere to and may therefore be an effective tool in cases where therapists otherwise would refer patients to more experienced colleagues. Additionally, BWRT may also function as a tool for therapists working with patients immediately following trauma exposure, such as accidents or crisis situations, or as an effective intervention to provide to patients in waiting lists, prior to receiving other forms of evidence-based treatments.

#### Cost-effectiveness

Even if our findings indicate that BWRT is inferior to TAU, the degree of inferiority should be considered from a cost-effectiveness point of view. Given that this is the case, appropriate analyses should then be conducted, taking into consideration the amount of time required for each condition, in respect to the potential effects of each therapeutic intervention. If our results indicate that participants experience alleviation of trauma symptoms following the BWRT intervention, despite showing inferiority to TAU, then BWRT might still hold the potential to function as a first line treatment.

### Limitations

Despite the outlined importance of conducting the proposed study, there are several limitations of the current protocol that need to be addressed, related to the generalizability of results, the primary and secondary outcome measures, lack of qualitative data and translation of the protocol.

#### Generalizability of results

The proposed study aims to include a heterogenous group of trauma victims, both regarding trauma experiences and comorbid conditions, in order to ensure greater generalizability of our results. However, this is not without limitations. Although we have taken important steps toward liberal inclusion and exclusion criteria to make the sample as representative of the general PTSD-population as possible, there are several challenges related to the generalizability of the results. In addition to accepted comorbid conditions, such as anxiety and depressive disorders, PTSD is also a relatively common underlying and comorbid condition in patients suffering from severe suicidality, psychosis and severe substance abuse [8]. Due to ethical concerns, these participants are excluded from the study sample, potentially leaving out an important sub-group of PTSD-patients. Therefore, the current design cannot inform whether or how patients with such comorbid conditions will respond to BWRT. Additionally, like many other clinical studies, our recruitment strategy is neither designed nor expected to recruit participants with severe PTSD, as these patients might not have the capacity to participate in studies and otherwise usually require and obtain immediate attention from the public health system in Norway. Taken together with the fact that we will include participants with subthreshold PTSD along with participants who meet the criteria for PTSD diagnosis, our sample will likely be skewed toward representation of the mild-to-moderate PTSD-population, and hence might not be generalizable to a broader population of trauma survivors suffering from more severe forms of PTSD.

#### Changes in primary and secondary outcome measures

Our primary measure of PTSD symptom severity relies on the CAPS-5, which only takes into account the operational definition of PTSD related to one (the most troublesome) traumatic event. Although we expect at least a portion of our participants to have experienced more than one traumatic incident, our design will not measure changes in PTSD-symptoms related to traumatic experiences not measured by the CAPS-5. However, our secondary measures might be more sensitive to measure overall symptom alleviation, although indirectly, through assessing variables known to be related to symptoms of PTSD, such as changes in perceived quality of life, rumination, as well as functional and cognitive abilities.

#### Lack of qualitative data

While our current design yields informative quantitative data related to specific predetermined outcomes, there are no qualitative measures, rendering the end results free of phenomenological perspectives. In other words, we will not be able to measure or describe any experiential processes of either therapy. This may leave out potentially important information regarding the mechanisms or phenomenology of the therapy process. Collection of qualitative data will be a valuable endeavor for future research should BWRT prove to be efficacious through preliminary quantitative investigations such as the proposed study.

#### Translation of protocol

The protocol is originally written in English for use with English speaking patients. In the proposed study, we will be using a translated version of the protocol which has been submitted to professional translators and subsequently evaluated and approved by Norwegian trained BWRT-therapists, but not yet tested in a Norwegian setting. Despite this, we consider this risk of bias related to translation to be low, as the protocol instructions are quite simple.

## Conclusion

To our knowledge, this study will be the first to systematically and empirically investigate the effectiveness of BWRT compared to standard evidence-based treatment. Given the similarity of BWRT to other proven treatment methods, existing evidence supporting the efficacy of other brief psychological treatments, as well as safety procedures provided in the current study protocol, we propose that the potential gains greatly outweigh the risks of the current trial. Our findings may contribute to important advances in psychological treatment of patients with subthreshold PTSD and PTSD, through making trauma treatment more accessible and battling current barriers to care. We therefore believe this investigation is crucial in order to obtain empirical data that can be used to inform whether BWRT has a future in the treatment of PTSD or not. We are confident that the proposed study represents an important first step in investigating the efficacy of BWRT.

### Trial status

The trial is approved by the Regional Committees for Medical and Health Research Ethics (REC) with trial identifier #191548, with registration date 23.04.2020. Protocol version 1.0, date: 01.12.2020. Approximate recruitment start: January 2022. Approximate completion of recruitment: December 2022

## Abbreviations

ART: Accelerated resolution therapy
BWRT: Brain working recursive therapy
BORA: Bergen open research archive
CAPS-5: The clinician administered PTSD scale for DSM-5
CBT: Cognitive behavioral therapy
CPT: Cognitive processing therapy
EMDR: Eye movement desensitization and reprocessing
LEC-5: Life event checklist for DSM-5
M.I.N.I.: The mini-international neuropsychiatric interview
NICE: National institute for health and care excellence
PC-PTSD-5: The primary care PTSD screen for *DSM-5*
PDQ-D5: Perceived deficits questionnaire-depression, 5-item
PE: Prolonged exposure
PTSD: Post-Traumatic stress disorder
RRS: Ruminative responses scale
SWLS: The satisfaction with life scale
TAU: Treatment as usual
TF-CBT: Trauma focused cognitive behavioral therapy
WSAS: Work and social adjustment scale
