# Correction to: A brief intervention for PTSD versus treatment as usual: Study protocol for a non-inferiority randomized controlled trial

**DOI:** 10.1186/s13063-021-05924-z

**Published:** 2021-12-16

**Authors:** Halvor Stavland, Camilla Refvik, Jarle Eid, Rafiq Lockhat, Åsa Hammar

**Affiliations:** 1 Faculty of Psychology, University of Bergen, Bergen, Norway; 2 Center for Crisis Psychology, University of Bergen, Bergen, Norway; 3 Cape Town, Summit Clinic, Cape Town, South Africa; 4 Department of Biological and Medical Psychology and Division of Psychiatry, Haukeland University Hospital, University of Bergen, Bergen, Norway

**Correction to: Trials 22, 737 (2021)**

**https://doi.org/10.1186/s13063-021-05674-y**

Following the publication of the original article [1], we were notified of the following corrections:

1. Under the headline BWRT:

**Incorrect**: A recent addition to the brief treatment arena for PTSD is Brain Working Recursive Therapy (BWRT®) [56], a single-session intervention based on an understanding of PTSD as being caused and maintained by maladaptive processing of the traumatic memory, in which the main goal of the therapy is to help the client change these maladaptive patterns in order to alleviate symptoms. The intervention is carried out following a strict protocol with a well-defined procedure, [56] without the patient having to disclose the full details of the traumatic memory.

**Correct**: A recent addition to the brief treatment arena for PTSD is Brain Working Recursive Therapy (BWRT®) [75], a single-session intervention based on an understanding of PTSD as being caused and maintained by maladaptive processing of the traumatic memory, in which the main goal of the therapy is to help the client change these maladaptive patterns in order to alleviate symptoms. The intervention is carried out following a strict protocol with a well-defined procedure, **originally developed by Terence Watts**, [56] without the patient having to disclose the full details of the traumatic memory.

2) In the section of authors contribution:

**Incorrect**: RL has been clinical advisor and is responsible for the development of the **original BWRT intervention protocol.**

**Correct**: RL has been clinical advisor and is responsible for the development of the **South African adaption of the original BWRT protocol**.
